# Tumour necrosis factor signalling in health and disease

**DOI:** 10.12688/f1000research.17023.1

**Published:** 2019-01-28

**Authors:** Jonathan Holbrook, Samuel Lara-Reyna, Heledd Jarosz-Griffiths, Michael F. McDermott

**Affiliations:** 1 Leeds Institute of Rheumatic and Musculoskeletal Medicine (LIRMM), Leeds, UK; 2 Leeds Institute of Medical Research at St. James’s, Leeds, UK; 3 Leeds Cystic Fibrosis Trust Strategic Research Centre, Leeds, UK

**Keywords:** TNF, TNFR, Cell death, Immunometabolism, Autoinflammatory

## Abstract

The master pro-inflammatory cytokine, tumour necrosis factor (TNF), has been shown to modulate multiple signalling pathways, with wide-ranging downstream effects. TNF plays a vital role in the typical immune response through the regulation of a number of pathways encompassing an immediate inflammatory reaction with significant innate immune involvement as well as cellular activation with subsequent proliferation and programmed cell death or necrosis. As might be expected with such a broad spectrum of cellular effects and complex signalling pathways, TNF has also been implicated in a number of disease states, such as rheumatoid arthritis, ankylosing spondylitis, and Crohn’s disease. Since the time of its discovery over 40 years ago, TNF ligand and its receptors, TNF receptor (TNFR) 1 and 2, have been categorised into two complementary superfamilies, namely TNF (TNFSF) and TNFR (TNFRSF), and 19 ligands and 29 receptors have been identified to date. There have been significant advances in our understanding of TNF signalling pathways in the last decade, and this short review aims to elucidate some of the most recent advances involving TNF signalling in health and disease.

## Introduction

Since the identification of tumour necrosis factor (TNF) in 1975 ^1^ and its isolation and characterisation in 1984 ^2^, the 17 kDa secreted form of this molecule has been established as a potent inflammatory cytokine with a myriad of diverse functions across a number of different cell types. Initially discovered as a serum factor which induced cell death in tumour cells ^1^ and thought at that time to be a promising target as a cancer treatment, TNF was later realised to be a potential target for the treatment of inflammatory diseases, such as rheumatoid arthritis (RA) ^3^, Crohn’s disease (CD) ^4^, ankylosing spondylitis (AS) ^5^, and psoriasis ^6^. However, localised administration of TNF is used as an effective therapy via isolated limb perfusion in patients with melanoma with multiple in-transit metastases ^7^.

TNF is produced primarily by monocytes/macrophages, but a number of other cell types, such as T and B lymphocytes, mast cells, natural killer cells, neutrophils, fibroblasts, and osteoclasts, can also secrete TNF ^8^, albeit in smaller quantities.

**Figure 1.**
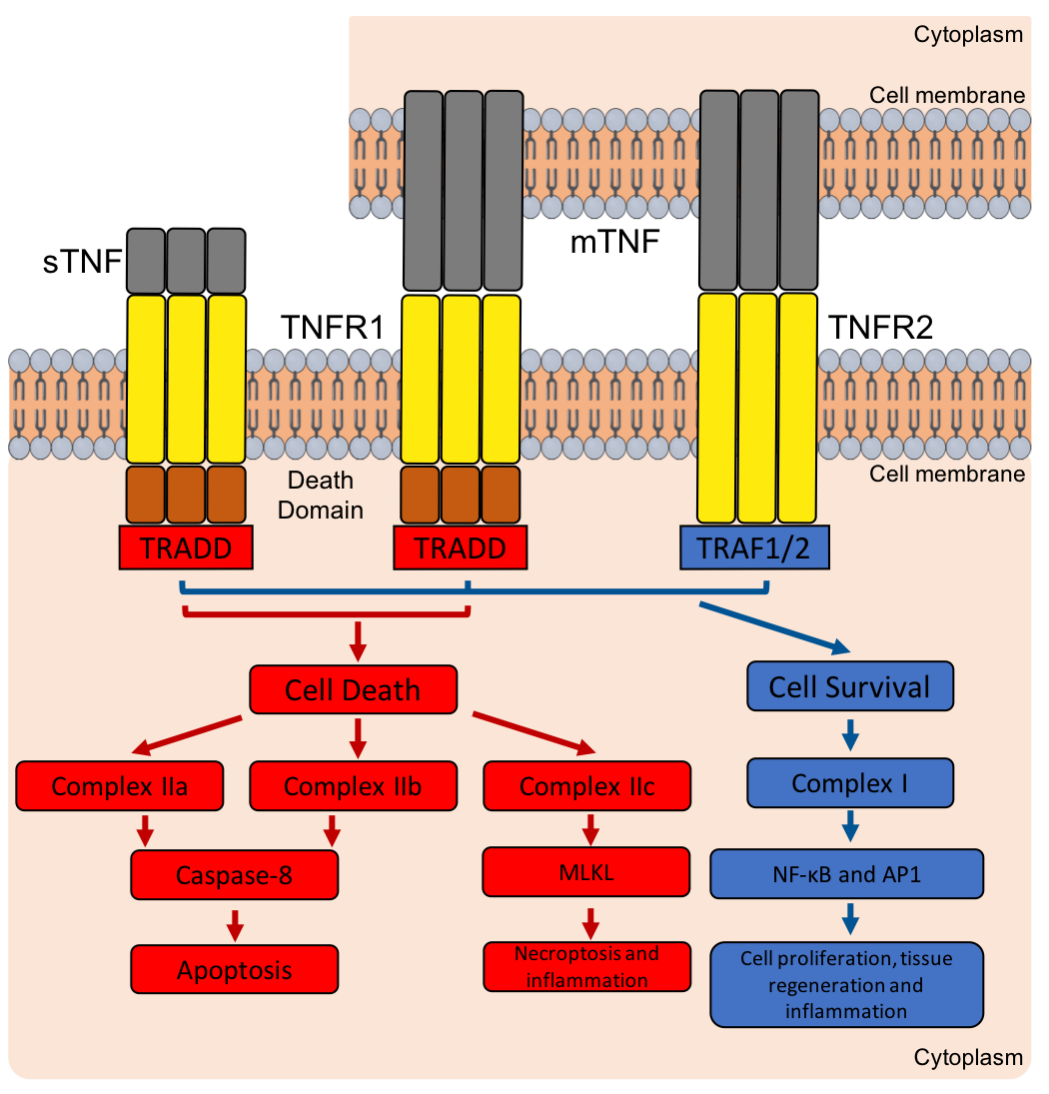
Overview of tumour necrosis factor (TNF): TNF receptor 1/2 (TNFR1/2) signalling pathways. Both soluble TNF (sTNF) and membrane TNF (mTNF) activate TNFR1. TNFR1 contains a death domain which interacts with TNFR1-associated death domain (TRADD). Depending on the ubiquitination state of receptor-interacting serine/threonine-protein kinase 1 (RIPK1), the cell undergoes apoptosis (via complexes IIa and IIb), necrosis (via complex IIc), or cell survival (via complex I). The formation of complexes IIa and IIb leads to the cleavage of pro-caspase-8 to form caspase-8 and induction of apoptosis. When complex IIc forms, the mixed lineage kinase domain-like protein (MLKL) is activated, inducing necroptosis. Upon the formation of complex I, cell survival is induced via the activation of nuclear factor-kappa B (NF-κB) and AP1 transcription factors because of RIPK1 ubiquitination. TNFR2, however, is activated primarily by mTNF and does not contain an intracellular death domain but interacts directly with TNFR-associated factor (TRAF) 1 and 2 to induce the formation of complex I with induction of homeostatic signals.

TNF is first produced as a 26 kDa 233-amino-acid transmembrane protein (mTNF) that is expressed on the cell surface, where it either continues to reside or is actively cleaved by TNF-converting enzyme to produce a 17 kDa 157-amino-acid soluble TNF (sTNF) form; sTNF is subsequently released and becomes detectable in the blood plasma ^9^. mTNF and sTNF both perform cellular functions mediated by either of its two receptors: TNFR1, expressed across all human tissues, and TNFR2, expressed primarily in immune cells, neurons, and endothelial cells ^10, 11^. mTNF functions as a ligand transmitting cell-to-cell interactions and, when bound to TNFR2 (its primary biological target) ^12^, is able to induce a more potent response than sTNF; interestingly, mTNF has also been shown to function as a receptor by initiating a cell signalling cascade through outside-to-inside signalling ^13^. TNFR1 and 2, though similar in their extracellular structures at the mTNF- and sTNF-binding sites, have distinct intracellular structures which bind to a number of adaptor proteins ^14^. TNFR1’s cytoplasmic tail contains a death domain (DD), thereby allowing it to recruit the TNFR1-associated DD (TRADD) ^15^; TNFR2, on the other hand, does not have an intracellular DD and recruits the TNFR-associated factor (TRAF) 1 and 2 proteins instead ( Figure 1) ^16^. Whereas both TNFR1 and 2 signalling pathways may lead to the activation of nuclear factor-kappa B (NF-κB) and the induction of a cell survival response, TNFR1 is also capable of inducing a cell death response depending on prevailing physiological circumstances; however, the regulation of the two TNFRs is dependent on the cellular environment and is not fully understood ( Figure 1) ^17^. The past decade has seen remarkable progress in the elucidation of regulatory cross-talk between TNFR1 and 2 as well as the individual but complementary functions of these two pleiotropic receptors ^18, 19^.

**Figure 2.**
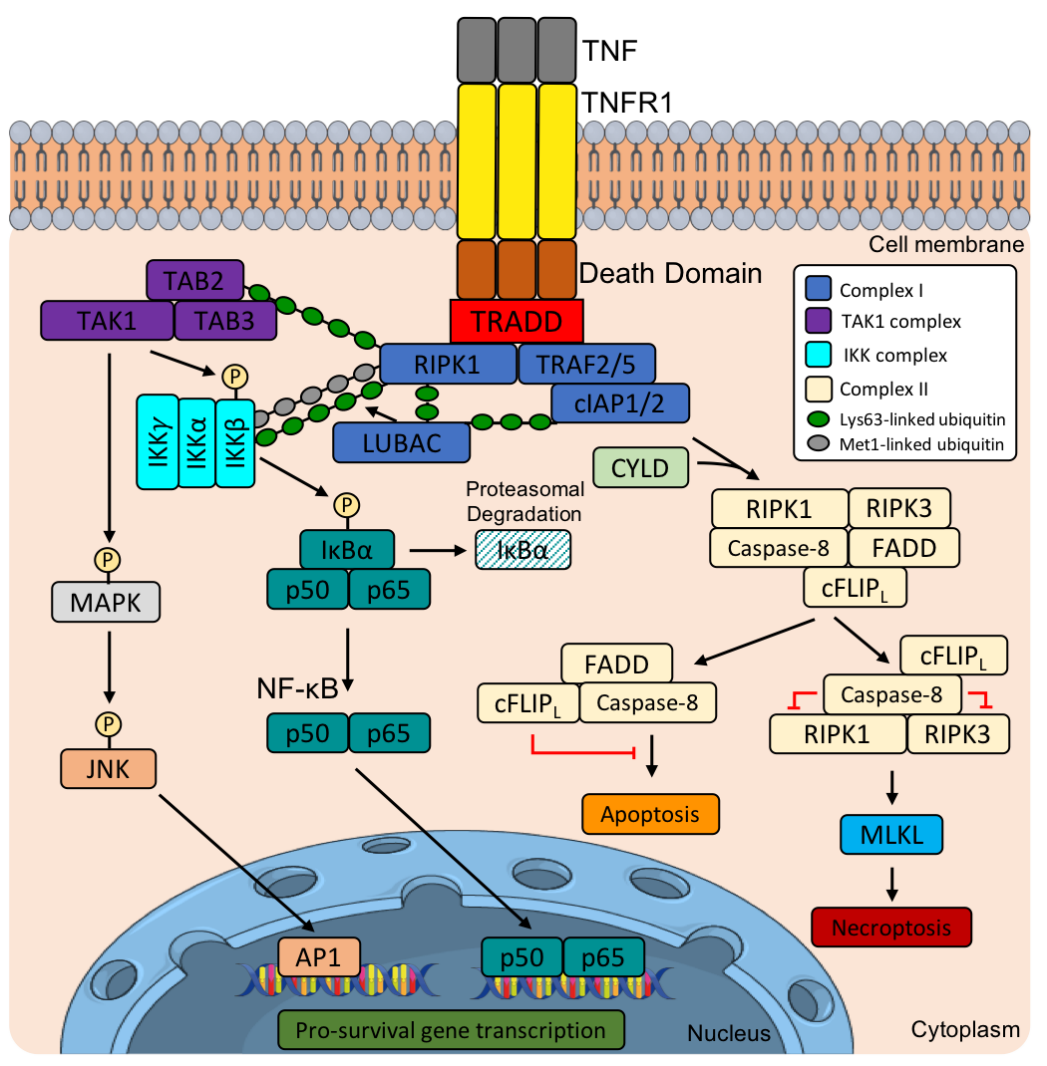
Overview of the tumour necrosis factor receptor 1 (TNFR1) signalling pathway. In the binding of TNF to TNFR1, TNFR1 recruits the TNFR1-associated death domain (TRADD), which then binds to receptor-interacting serine/threonine-protein kinase 1 (RIPK1), TNFR-associated factor 2 or 5 (TRAF2/5), and cellular inhibitor of apoptosis protein 1 or 2 (cIAP1/2) to form complex I. cIAP1/2 and the linear ubiquitin chain assembly complex (LUBAC), consisting of HOIP, HOIL, and SHARPIN, add Met1-linked and Lys63-linked polyubiquitin chains, respectively, to RIPK1. This stabilises RIPK1, amplifying its signal. Lys63-linked chains on RIPK1 recruit the transforming growth factor-beta (TGFβ)-activated kinase 1 (TAK1) complex, consisting of TGFβ-activated kinase 1 and mitogen-activated protein kinase (MAPK)-binding protein 2 and 3 (TAB2 and 3) and TAK1. The TAK1 complex phosphorylates MAPK, Jun N-terminal kinase (JNK), and the IκB kinase (IKK) complex. This results in the translocation of transcription factors AP1 and nuclear factor-kappa B (NF-κB) into the nucleus, leading to the transcription of target genes. RIPK1 is deubiquitinated by cylindromatosis tumour suppressor protein deubiquitinase (CYLD), facilitating its dissociation from complex I to form complex IIb, consisting of RIPK1, RIPK3, FAS-associated death domain (FADD), caspase-8, and FLICE-like inhibitory protein long (cFLIP _L_). cFLIP _L_ regulates both the apoptosis and the necrosis pathway, preventing caspase-8 activation to avert apoptosis. cFLIP _L_ acts in complex with caspase-8 to cleave RIPK1 and RIPK3 to inhibit their aggregation and the activation of mixed lineage kinase domain-like protein (MLKL), which would result in necrosis. HOIL-1, heme-oxidised iron regulatory protein 2 ubiquitin ligase 1; HOIP, HOIL-1 interacting protein.

Upon binding of TNF to TNFR1, TNFR1 undergoes a conformational change in its DD, whereby both TRADD and receptor-interacting serine/threonine protein kinase 1 (RIPK1) are recruited, leading to the formation of complex I and the initiation of cell survival via activation of the NF-κB pathway ( Figure 2) ^20– 22^. TNF-mediated cell death is highly dependent on various cell death check points, which regulate the pro-survival pathway; when these check points are disrupted, TNF-mediated signalling occurs through the formation of complexes IIa, IIb, and IIc ^23^. The ubiquitination status of RIPK1 is a critical determinant of whether a particular signalling pathway promotes cell survival, through complex I ^24^, apoptosis (controlled cell death), via complex IIb ^25, 26^, or necrosis (uncontrolled cell death), through complex IIc ^27, 28^.

TNF signalling is tightly regulated by post-translational ubiquitination, an essential mechanism for the regulation of many biological processes. Ubiquitin (Ub) chains are assembled in response to activation of the TNF receptors and then attached to target substrates to modulate protein function. The type of Ub chains is determined by their specific linkage type, which are assembled to generate distinct intracellular signals. Deubiquitinase (DUB) enzymes reverse the process of ubiquitination by hydrolysing Ub moieties from the modified protein substrates ^29^. Ub retains seven lysine sites (K6, K11, K27, K29, K33, K48, and K63) at the N-terminal methionine (Met-1) and the C-terminal glycine site. The main sites of interest are K48 and K63, as they are essential for the activation of the NF-κB pathway ^30^.

The activation of complex I relies on the ubiquitination of RIPK1 and consists of TRADD ^15^, RIPK1 ^21^, TRAF2 or 5 ^31^, cellular inhibitor of apoptosis protein (cIAP) 1 or 2 ^32^, and linear Ub chain assembly complex (LUBAC) ( Figure 2) ^33^. LUBAC is composed of three proteins: heme-oxidised iron regulatory protein (IRP) 2 Ub ligase 1 (HOIL-1), shank-associated RH domain-interacting protein (SHARPIN), and HOIL-1 interacting protein (HOIP). cIAP and LUBAC add K63-linked and Met1-linked polyUb chains, respectively, to RIPK1, stabilising the Ub structure and amplifying its signal ^33– 35^. This leads to the recruitment of transforming growth factor-beta (TGF-β)-activated kinase (TAK) 1 complex, consisting of TAK-binding protein (TAB) 2 and 3, and inhibitor of κB (IκB) kinase (IKK) complex, composed of the NF-κB essential modulator (IKKγ, also known as NEMO), IKKα, and IKKβ ^24^. The TAK1 complex phosphorylates mitogen-activated protein kinase (MAPK), leading to a signalling cascade whereby c-Jun N-terminal kinase (JNK), p38, and AP1 transcription factors are activated, and the IKKβ activates NF-κB. This ultimately leads to pro-survival signalling, where inflammation and the proliferation of immune cells are induced ^24^. Following the activation of NF-κB, cellular FLICE-like inhibitory protein long (cFLIP _L_) translocates to complex IIa in order to prevent caspase-8 activation. However, if the late NF-κB-dependent check point is disrupted and cFLIP _L_ levels are consequently reduced, apoptosis is initiated via complex IIa (composed of TRADD, FAS-associated DD [FADD], and pro-caspase-8) through auto-activation of pro-caspase-8 ^36– 38^. In contrast to the late check point, the early check point, which occurs immediately after ligand binding, is initiated by the ubiquitination of RIPK1 by cIAP and LUBAC ( Figure 2) ^39– 41^.

When RIPK1 is not ubiquitinated, complex IIb is formed; in order for this to occur, the cylindromatosis tumour suppressor protein DUB (CYLD) enzyme deubiquitinates RIPK1, thereby allowing it to disassociate from complex I ^42^ and form complex IIb, whereby TRADD is replaced by RIPK3, upon degradation of cIAP1 and 2 ^25, 26, 43^. This, in turn, leads to the cleavage of pro-caspase-8 to caspase-8, and activation of the caspase signalling cascade results in apoptosis ( Figure 2). To ensure that apoptosis, and not necrosis (unregulated cell death), is induced, which could lead to unwanted inflammation and damage to the surrounding tissues, caspase-8 or pro-caspase-8 in complex with cFLIP _L_ is required to cleave RIPK1 and 3 ^25, 44^. However, RIPK1 and 3 may, on occasions, remain uncleaved, leading to their aggregation and the formation of complex IIc, with the resultant activation of mixed lineage kinase domain-like protein (MLKL) and the induction of a regulated form of necrotic cell death, necroptosis ( Figure 2) ^27, 28^.

TNFR2 interacts directly with TRAF1 or 2 to recruit cIAP1 or 2 to promote cell survival signalling through the formation of complex I and the induction of NF-κB, MAPK, and Akt, promoting cell proliferation and tissue regeneration. The binding of TRAF2 to TNFR2 is considerably weaker than TRAF2’s binding to TRADD ^45, 46^, indicating a regulatory role of the TNFR1 signalling pathway ^45, 47^. It is hoped that, through greater understanding of TNF signalling in health and disease, improved therapies can be developed to inhibit this potent cytokine in a more target-specific manner.

Throughout the TNF signalling pathway, there are a number of proteins that are essential for negative regulation of the pathway. A20 contains both DUB and E3 ligase domains and plays a vital regulatory role at multiple steps of the TNF signalling pathway, such as its removal of K48- and K63-linked Ub chains from RIP1 through its zinc finger (ZF) 4 domain ^48, 49^ and its inhibition of the interaction between LUBAC and IKKγ through binding to their linear Ub chains via A20’s ZF7 domain upon TNF stimulation ^50, 51^. A20, as well as the regulatory molecule TAX1BP1, has also been shown to inhibit the E3 ligase activities of TRAF2, TRAF6, and cIAP1 by interfering with E2 Ub enzymes Ubc13 and UbcH5c, disrupting NF-κB signalling ^52^. In 2015, it was documented, in a mouse embryonic fibroblast (MEF) model, that binding of A20 to linear Met1-linked polyUb chains protected the MEFs from CYLD-mediated degradation. This was shown to be dependent on A20’s ZF7 domain but was independent of its DUB activity and protected cells from TNF-induced RIPK1-dependent apoptosis (RDA) ^53^. Another DUB, ovarian tumour (OTU) DUB with linear linkage specificity (OTULIN), acts via its specific cleavage of Met1-linked polyUb chains after it binds to the HOIP component of the LUBAC ^54^. CYLD is another negative regulator of the TNF signalling pathway, removing Ub chains from several proteins such as TRAF2, TRAF6, IKKγ, and RIPK1 to regulate the NF-κB and JNK pathways ^55^.

## Cell death

The ubiquitination status of RIPK1 is a critical determinant of whether a cell undergoes RDA; a recent study describes a “detergent insoluble, highly ubiquitinated and activated RIPK1 pool” ^56^, termed iuRIPK1, which acts as an intermediate between complex I and cytosolic complex IIb formation and caspase activation. Using a systematic screen for RDA, the investigators found that iuRIPK1 is regulated by Parkinson’s disease-associated leucine-rich repeat kinase 2 (LRRK2), E3 Ub ligase, c-Cbl, and ALS-associated NEK1, suggesting a mechanistic link between RDA and neurodegenerative conditions ^56^. It is well documented that, in addition to its Ub status, kinase activity of RIPK1 is essential for RDA and necroptosis, both being induced by TNF ^57– 59^. In 2018, Meng *et al*. reported that C-terminal DD dimerisation of RIPK1, via K584, serves as an amplification mechanism to promote RIPK1 kinase activity and that increased expression, under pathological conditions, may promote its dimerisation and activation ^60^. In light of these discoveries, the question arises as to whether detergent-insoluble lipid rafts are providing a platform for the assembly of cell surface signalling complexes, under certain pathological conditions, and thus might act as a critical determinant in driving RIPK1-mediated cell death ^61^. Recent research has also found that RIPK1 prevents skin inflammation, via the inhibition of RIPK3-MLKL-dependent necroptosis, mediated by the cytoplasmic DNA sensor protein, Z-DNA-binding protein (ZBP1) ^62, 63^, and that deficiency of RIPK3, as well as an inactive form of RIPK3, reduces necroptosis and thereby the severity of inflammation in mouse models of tissue injury ^64^.

Controversy hangs over the role of A20 DUB, which has been shown in separate studies to either protect or promote TNF-mediated RDA ^53, 65^. A recently published report by Garcia-Carbonell *et al*. ^65^ reported increased levels of *A20* gene expression in patients with irritable bowel disease (IBD), and intestinal epithelial cells have increased susceptibility to TNF-induced cell death. Similarly, stabilising the function of *A20*, in the form of homodimers, facilitated ripoptosome complex (consisting of RIP1 and cIAP1 and 2) assembly by binding linear Ub chains via ZF7, protecting RIPK1 from deubiquitination, resulting in enhanced caspase-8 recruitment and activation ^65^. A20 has also been shown to protect cells from necroptosis through the deubiquitination of RIPK3 in both T cells and fibroblasts ^66^.

Alongside their functional roles as E3 Ub ligases, the cIAPs are critical regulators of pro-inflammatory signalling pathways ^67^. The loss of IAP induces not only the formation of complex IIb and stimulates RDA but also caspase-8-dependent interleukin-1 beta (IL-1β) maturation and NLRP3 inflammasome signalling in macrophages, in a TLR4/TRIF-dependent manner which is independent of TNFR1 ^68, 69^. Blocking caspase-8 activity, followed by interferon gamma (IFNγ) priming, rendered neutrophils sensitive to TNFR1-dependent necroptosis via the RIPK3-MLKL pathway, leading to NLRP3 activation ^70^. The finding that cell death pathways are distinctly regulated in neutrophils, as compared with other myeloid cells, signifies the importance of cell type on immune signalling pathways in orchestrating a co-ordinated inflammatory response.

## Cellular activation and proliferation

TNF is known to have widespread and profound effects on both the activation and the proliferation of different subsets of immune cells in several disease states. *In vitro* anti-TNF blockage, used in T-cell monocyte co-cultures of patients with the autoimmune disorder thrombocytopenia, produced a robust proliferation of the immunomodulatory regulatory T (Treg) cells ^71^; interestingly, this Treg cell expansion was dependent on TNFR2 and not TNFR1. Blockage of TNFR2 resulted in a robust expansion of Treg cells, whereas neutralisation of TNFR1 had no effect on this Treg cell expansion ^71^. Therefore, TNFR2 might be considered a potential novel therapeutic target for immunomodulation, not only in thrombocytopenia but also in other unrelated immune disorders associated with decreased levels of Treg cells, such as RA, AS, systemic lupus erythematosus (SLE), IBD, and psoriasis ^72^. For a recent review of anti-TNFR2 therapy, see Zou *et al*. ^73^.

Another report showed that inhibition of TNF signalling, by a number of anti-TNF biological treatments, primed naïve CD4 ^+^ T cells towards a regulatory phenotype with high expression of IL-10 and reduced IFNγ production ^74^. Patients with cardiovascular disease often exhibit high levels of blood TNF, which are positively correlated with the formation of foam cells, and there are further repercussions for the development of plaques and blood clots ^75^. These observations were supported by both *in vitro* and *in vivo* experiments that confirmed that high levels of TNF enhanced the expression of adhesion molecules and scavenger receptors on blood monocytes ^75^. TNF has an important role, not only in immune cells but also in the regulation of circadian rhythms by the central nervous system. One study reported that TNF stimulation of the suprachiasmatic nucleus exerted an important influence on the regulation of circadian rhythms, through the activation of TNFR1 after lipopolysaccharide (LPS) inoculation, mainly during the early period of the night, when TNFR1 showed its highest expression ^76^. This regulation has novel implications for several disorders and might explain some of the observed disruption of circadian rhythms during disease ^76– 78^, perhaps due to higher expression of TNF in activated immune cells.

A20 has been shown to promote cell survival of CD4 T cells by initiation of autophagy via its inhibition of mammalian target of rapamycin (mTOR) ^79^ as well as to restrain the development of Treg cells, as A20-deficient mice present with enlarged thymic and peripheral Treg cell compartments ^80^. A20 has also been shown to exert an important defence role against bacterial infections, as it enhances secondary CD8 ^+^ T-cell responses but reduces the primary response ^81^. SHARPIN, a component of LUBAC, has a number of modulating effects on T cells; for example, defective SHARPIN results in a significant reduction in the overall population of Treg cells and their ability to function correctly ^82^. Furthermore, deficiency of SHARPIN leads to reduced numbers of CD4 ^+^ CD25 ^+^ FOXP3 ^+^ Treg cells in the blood, spleen, lymph nodes, and thymus ^83^. HOIL-1, which is another component of LUBAC, has been reported to be cleaved by mucosa-associated lymphoid tissue lymphoma translocation 1 (MALT1), leading to its becoming a potent inhibitor of LUBAC-induced NF-κB signalling in activated T cells ^84– 87^; other NF-κB regulatory proteins that are cleaved by MALT1 include A20 ^88^, RelB ^89^, and CYLD ^90^, not to mention the auto-proteolytic cleavage of MALT1 ^91^.

A recent study focusing on innate immune cells showed the importance of TNF activation of these cells in cerebral tuberculosis, although neuron-derived TNF also plays a limited role ^92^. TNF has a ubiquitous influence on different cells and tissues and has an important role in the tumour microenvironment. A recent publication reported that regulation of the immunomodulatory check point programmed death-ligand 1 (PD-L1) in tumour-associated macrophages and monocytes was strongly increased by TNF in a B16 melanoma mouse model of disease ^93^. By using TNFR ^–/–^ mice (strain B6.129 S-Tnfrsf1a ^tm1Imx^ Tnfrsf1b ^tm1Imx^/J), the researchers found a significant decrease in numbers of tumour-associated macrophages and dendritic cells expressing PD-L1 and an associated reduction in the size of the tumours ^93^. In a separate study, TNF was reported to activate the NF-κB signalling pathway and upregulate PD-L1 in human prostate and colon cancer cells, thereby promoting immunosuppression and favouring the tumour microenvironment ^94^. Immunotherapy has proven to be an effective option in the treatment of several cancers with high expression of PD-L1; thus, incorporating anti-TNF biologics into this therapeutic regimen may result in improved outcomes for certain types of cancers.

## Immunometabolism

The link between metabolism and immunity first became apparent in the 1980s, when macrophage-conditioned media, stimulated with LPS, was found to increase lipoprotein lipase expression and promote resistance to insulin in adipocytes ^95, 96^. It was later observed that obese rats expressed increased levels of TNF in their adipose tissue and that obese humans also expressed TNF at higher levels in their muscle tissue ^97– 99^. In obese rats, TNF inhibition led to improved metabolism of glucose and improved insulin sensitivity ^100, 101^; however, when TNF was administered, the opposite effects occurred ^102, 103^.

The essential glycolytic enzyme, glyceraldehyde 3-phosphate dehydrogenase (GAPDH), under low glycolysis conditions has been linked to regulation of the inflammatory response, as it binds to TNF mRNA in monocytes and macrophages and post-transcriptionally inhibits its production ^104, 105^, thus showing that the metabolic state of immune cells helps regulate various immune responses. Additionally, the product of GAPDH, nicotinamide adenine dinucleotide (NADH), significantly inhibited TNF secretion in macrophages ^105^.

The anti-inflammatory metabolite itaconate was found to inhibit TNF in an LPS-stimulated mouse model as well as in human peripheral blood mononuclear cells; however, no effects were observed in human macrophages ^106^. Immature monocyte-derived dendritic cells have a high expression of the extracellular succinate sensor, a G-protein-coupled receptor known as GPR91, and these cells were shown to have enhanced production of TNF due to succinate when stimulated with the TLR7 ligand imiquimod or the TLR3 ligand polyinosinic-polycytidylic acid (poly[I:C]) ^106^. Macrophages have also been recognised to upregulate their expression of GPR91 and release succinate into the extracellular milieu under inflammatory signals, which may lead to exacerbations of RA, as rheumatoid joints have high levels of succinate present ^107^.

Cis-aconitate decarboxylase (CAD), the enzyme product of immune-responsive gene 1 (IRG1), catalyses the decarboxylation of cis-aconitate to produce itaconate, and this reaction is induced by heme oxygenase 1 (HO-1) activity. Carbon monoxide induction of HO-1 led to reduced TNF levels via upregulated IRG1 expression, demonstrating IRG1’s anti-inflammatory effects ^108^. Another glycolytic enzyme, alpha-enolase (ENO1), has been shown to have inflammatory effects when expressed on the surface of monocytes and macrophages by contributing to the production of inflammatory cytokines, such as TNF, in the synovium of patients with RA and also in the type II collagen model of arthritis ^109^. ENO1 also plays a role in both the induction and the function of Treg cells via the promotion of Foxp3 splicing ^110^.

A recent study showed that inhibition of complex II of the mitochondria reduces the production of TNF in macrophages ^111^, and another study demonstrated the importance of mitochondrial citrate carrier (CIC) in TNF-triggered inflammation, whereby the CIC is transcriptionally upregulated under TNF stimulation via NF-κB and plays an essential role in regulating the downstream production of nitric oxide and prostaglandins ^112^. In macrophages, citrate accumulation is essential for fatty acid synthesis, in a murine model of type 2 diabetes, which enhances inflammatory signalling through TNF production ^113, 114^.

## Tumour necrosis factor signalling-related inflammatory disease

Although TNF plays an essential role in health, it also exerts a double-edged sword effect in several disease states. Indeed, in 1999, mutations in the *TNFRSF1A* gene, encoding TNFR1, were found to be associated with episodic fevers and profound localised inflammation; consequently, TNFR1-associated periodic syndrome (TRAPS) was defined as one of the disease entities under the collective heading of autoinflammatory diseases ^115^. The term “autoinflammation” was used to refer to the increasing number of clinical disorders characterised by episodes of seemingly unprovoked inflammation in the absence of high autoantibodies titres or antigen-specific T lymphocytes. Anti-TNF therapy was used for the treatment of TRAPS with some initial success; however, a large number of these patients eventually become resistant to this treatment ^116, 117^, and an increasing number of reports subsequently revealed the clinical benefits of anti-IL-1β and/or anti-IL-6 therapy in the forms of canakinumab and tocilizumab, respectively ^118– 123^. The current standard treatment for the more severe cases of TRAPS is IL-1β blockade.

Recently, the scope of autoinflammation has been broadened to encompass some abnormalities of deubiquitination and redox homeostasis under the name of ubiquitination-associated autoinflammatory diseases (UADs). There are a number UADs, but only two are known to directly affect the TNF signalling pathway, namely otulipenia/OTULIN-related autoinflammatory syndrome (ORAS) and A20 haploinsufficiency (HA20) ^124^. The DUB OTULIN is an essential negative regulator of inflammation and prevention of autoimmunity involving the TNF pathway. Genetic alterations of DUBs have been associated with neurodegenerative diseases and cancers ^125^; also, two unrelated monogenic systemic inflammatory diseases have been associated with defective DUBs ^124^. Otulipenia is caused by loss-of-function mutations in the linear (Met-1) OTULIN with resulting dysregulation of the deubiquitination process ^126, 127^. Increased signalling in the canonical NF-κB pathway with overproduction of TNF, IL-1β, IL-6, IL-12, IL-18, and IFNγ in response to LPS stimulation was reported in these patients. Anti-TNF therapy was found to normalise markers of active inflammation, including C-reactive protein (CRP) and erythrocyte sedimentation rate (ESR), and control disease activity in otulipenia ^124^.

Loss-of-function mutations in the *TNFAIP3* gene, which codes for A20, result in the UAD disorder, HA20, so named because of loss of function of one of the copies of the *A20* gene, leading to early onset systemic inflammation and a disorder resembling Behçet’s disease ^128^; for a recent review of HA20, see Kadowaki *et al*. ^129^. Polymorphisms in TNFAIP3 have also been linked to RA, SLE, psoriasis, and CD susceptibility through genome-wide association studies ^128, 130– 136^. A20 function has also been associated with a protective role in RA, as it negatively regulates NLRP3 inflammasome activity ^137^.

Keratinocyte-specific deletion of the LUBAC components HOIP or HOIL-1 results in a lethal inflammatory skin disease, caused by TNFR1-induced caspase-8-mediated apoptosis, that occurs independently of RIPK1 kinase activity ^138^. This differential effect in cell death pathway aetiology provides clues to the benefits of various treatment pathways. Interestingly, patients with LUBAC-inactivating germline mutations, such as HOIL-1 and HOIP mutations, also have primary immunodeficiency and autoinflammation ^139– 141^. Expression of TNFR1 in peripheral blood mononuclear cells from patients with RA has been positively correlated with the efficacy of therapies received ^142^; furthermore, the expression of both TNFR1 and 2 is downregulated in B cells of patients with RA compared with healthy controls ^142^.

## Summary

The identification of TNF and the development of anti-TNF therapies for associated disorders were revolutionary moments in disease research, resulting in vast improvement in the quality of life for patients with these debilitating diseases. With the ever-expanding understanding of TNF and its signalling pathways, it is hoped that the new research focus on combination therapies, and not just anti-TNF, will further improve these patients’ quality of life.
